# Serologic autoimmunologic parameters in women with primary ovarian insufficiency

**DOI:** 10.1186/1471-2172-15-11

**Published:** 2014-03-10

**Authors:** Xiumei Zhen, Jie Qiao, Rong Li, Lina Wang, Ping Liu

**Affiliations:** 1 Department of Obstetrics and Gynecology, Peking University, Third Hospital, No. 49 HuaYuan North Road, HaiDian District, Beijing 100191, China; 2 Key Laboratory of Assisted Reproduction, Ministry of Education, Beijing, China; 3 Beijing Key Laboratory of Reproductive Endocrinology and Assisted Reproduction, Beijing, China

**Keywords:** Primary ovarian insufficiency, Antibody, Immune, Microarray, Premature ovarian failure

## Abstract

**Background:**

Primary ovarian insufficiency (POI) is heterogeneous disease defined by amenorrhea or premature depletion of ovarian follicles before the age of 40 years. The etiology of POI is still unclear. The purpose of this study is to evaluate whether women with POI have an elevated serum levels of autoimmunologic parameters.

**Methods:**

The serum from peripheral blood samples which come from 96 POI patients and 100 age-matched health women were analyzed for a series of autoimmune antibodies using protein microarray. The antibodies to double-stranded DNA (ds-DNA), histone (HIS), nuclear ribonucleoprotein (RNP), Sjogren’s syndrome A (SSA/Ro), Sjogren’s syndrome B (SSB/La) and Smith antigen, Jo-1, scleroderma-associated antigen (Scl-70) and centromere (CEN), zona pellucid (ZP), adrenocortical antibodies (ACA),Rheumatoid factor (RF), glomerular basement membrane (GBM), proliferating cell nuclear antigen (PCNA), myeloperoxidase (MPO), proteinase 3 (PR3), thyroid microsomal antibody and antinuclear antibody (ANA)were analyzed.

**Results:**

Among the 96 women with POI and 100 age-matched health controls, women with POI had significantly elevated circulation levels of Jo-1 and PR3 (p = 0.010 and p = 0.001) whereas circulation levels of ANAs, dsDNA, histone, RNP, Sm, Scl-70, SSA, SSB, CEN, ZP, ACA, RF, GBM, PCNA, MPO and TM antibodies were similar between the two groups.

**Conclusions:**

This study shows that the autoimmune antibodies JO-1 and PR3 were significantly higher in POI women group which suggested that these antibodies may have played special role in POI, but the evaluation of the exact pathways of them remains to be determined.

## Background

In the normal course, a woman enters menopause at a mean age of 51 years (41-60 years). However, early menopause occurs approximately 1% of women before 40 years of age, approximately 0.1% of women under 30 and 0.01% of women younger than 20. This condition is termed as premature ovarian failure (POF), also referred to as primary ovarian insufficiency (POI), characterized by amenorrhea, sex steroid deficiency and elevated levels of gonadortropins. The etiologies include genetic causes, enzymatic and gonadotropin defects, ovarian insults, and autoimmunity. Approximately 20-30% of the cases with POI have associated autoimmune disorders [1]. A variety of auto-immune conditions such as endocrinopathy, thyroid diseases, addision’s disease, rheumatoid arthritis and polyglandular syndrome have been found in POI women.

A number of autoimmunologic diseases exhibit circulating characteristics. Antibodies to thyroid microsomal [2] and myeloperoxidase (MPO) are associated with graves’ disease and thyroiditis [3], antinuclear antibodies (ANA) and rheumatoid factor(RF) represent a serology hallmark in the diagnosis of systemic autoimmune rheumatic disease (SARD) such as systemic lupus erythematosus [4]. Zona pellucida antibodies are associated with POI, antibodies to double-stranded DNA (ds-DNA), histone, nuclear ribonucleoprotein (RNP) and Smith antigen [5] are associated with SLE, whereas antibodies to Sjogren’s syndrome A (SSA/Ro) and Sjogren’s syndrome B(SSB/La) can occur in both SLE and Sjogren’s syndrome (SS). Antibodies to MPO and proteinase 3 (PR3) have been demonstrated to mediate anti-neutrophil cytoplasmic antibody (ACNA)-associated disease which is the target of anti-neutrophil cytoplasm antibodies in granulomatosis with polyangiitis [6]. Antibodies to Jo-1 was observed in polymyositis and dermatomyositis, whereas antibodies to scleroderma-associated antigen (Scl-70) and centromere can occur in patients with progressive systemic sclerosis (PSS) [7]. Anti-glomerular basement membrane antibody is associated with glomerulonephritis [8]. Anti-RNP antibodies are linked with mixed connective tissue disease (MCTD) and SLE, anti-PCNA antibodies have been described in a variety of SARD.

The aim of the present study was to investigate a panel of serologic autoimmunologic parameters in POI women and contributed further data on autoimmunologic processes and to elucidate potential mechanisms for their interrelation in POI.

## Methods

### Selection criteria of patients and controls

A total of 96 patients and 100 controls were included in this study. A written consent was obtained from all patients and controls for their participation in the study. All of the procedures were carried out in compliance with the Declaration of Helsinki. This study was approved by the Peking University Third Hospital ethical committee (IRB00006761-2011022, about the details of the ethics statement please see the Additional file 1).

The inclusion criteria were (1) age <40 years, (2) amenorrhea for > 6 months, (3) FSH > 40IU/L (two times apart more than one month), (4) chromosome Karyotype is 46 XX. Control subjects were enrolled by the criteria (1) age-matched with the POI group, (2) menstrual rules, (3) hormone level was normal. None of them had a personal or family history of hereditary disease and pelvic surgery and no radiation and chemotherapy histories (such as previous pelvic irradiation, operative castration, and previous cytotoxic chemotherapy were excluded).

Blood was collected from the recruited individuals, serum separated, stored at -20°C till further use.

### Detection of antibodies

A protein microarray-based detection kit (CapitalBio, Beijing) was used, 18 antigens related with the serum autoimmune antibodies were immobilized on the microarray. The principle is to carry out a relative quantitative detection on the related antibodies using an indirect immunofluorescence assay on the chip. First of all, on-chip fixed antigens were reacted with lgG antibodies in the serum sample to form antigen - antibody complexes. The quantity of the antigen - antibody complex depends on the amount of specific lgG antibodies in serum. Then add excessive amount of Cy3 fluorescent labeled anti-human lgG antibody (secondary antibody) to the microarray which will interact with the complex to form the antigen-antibody-fluorescent-antibody complex, and the amount of the antigen-antibody-fluorescent-antibody complex on the array depends on the amount of antigen-antibody complex. The microarray was scanned by LuxScan 10K-B microarray scanner to detect the fluorescence signals on the array to form an image. Finally, the data was analyzed by the interpreted software on the machine and the serum concentrations of specific antibody were calculated.

Data was analyzed using a cutoff value to determine an antibody positive result. The cutoff value was equivalent to the mean value for the serum assay controls (n = 100) plus 2SD (95% CI) or 3SD (99% CI).

### Statistical analysis

Quantitave data were expressed as mean ± SD, T-test and chi-square or Fisher exact test were used to compare hormone serum levels, BMI, ovarian volume and antibodies. All tests applied two-tailed, and the significance level was defined as *p* value < 0.05. Statistical analysis was performed with the SPSS 11.5 package.

## Results

Among the 96 patients, 9 patients had histories of Mumps, 5 patients had histories of tuberculosis, 4 patients had histories of thyroid disorders and 2 patients had histories of diabetes mellitus. 10 of the patients presented with primary amenorrhea, 86 patients presented with secondary amenorrhea. The characters of POI and healthy women were seen in Table 1.

**Table 1:** Characteristics of the POI and health control women

**Parameter**	**POI (Range) N = 96**	**Health (Range) N = 100**
Age (year)	27.6 ± 4.51 (21-38)	27.5 ± 4.57 (21-39)
Menarche (age)	13.5 ± 1.71 (12-18)	13.4 ± 1.15 (12-15)
Amenorrhea (year)	6.33 ± 4.71 (2-18)	0
BMI (kg/m2)	20.5 ± 2.12 (16-27)	20.33 ± 1.56 (18-25)
FSH (IU/L)	80.25 ± 33.6 (44.7-140.2)*	7.6 ± 1.74 (5.35-9.7)
LH (IU/L)	38.65 ± 14.8 (20.4-89.7)*	4.29 ± 1.89 (2.89-9.56)
E2 (pmol/L)	80.4 ± 13.3 (73.4-109)*	136 ± 53.9 (96.9-240)
T (nmol/L)	0.781 ± 0.29 (0.69-1.28)**	1.32 ± 0.379 (1.21-2.19)
A (nmol/L)	5.46 ± 1.9 (2.5-6.7)	6.02 ± 1.97 (4.3-8.9)
Ovarian volume (ml)^#^	1.512 ± 2.01 (0.489-7.53)*	13.1 ± 1.61 (9.63-19.8)

*p = 0.000, **p = 0.002, ^#^ovarian volume was the average of two side of ovary volume, ovarian volume were calculated using the formula for a prolate ellipsoid: Longitudinal diameter × anterioposterior diameter × transverse diameter × 0.5233.

When a cutoff value (95% CI) was used, the number of positive PR3 and Jo-1 antibody were significantly higher in women with POI (15/96 & 3/100, *p* = 0.002 and 12/96 & 3/100, *p* = 0.012) whereas the circulation levels of ANAs, dsDNA, ssDNA, histone, RNP, Sm, Scl-70, CEN, ZP, AC, RF, GBM, PCNA, MPO and TM antibodies were similar between the two groups. A higher cutoff value (99% CI) reduced the proportion of positive antibodies. Using the higher cutoff value, PR3 and Jo-1 antibodies from the women with POI remained significantly different from antibodies from Control group (Table 2).

**Table 2:** Summarize of the laboratory findings in women characterized as autoimmune patients#

**Antibodies**	**POI Women (n = 96)**	**Healthy women (n = 100)**	***P *** **value**
SSA (%)	4.2 (4/96)	2 (2/100)	0.322
Sm (%)	0	0	--
SCL70 (%)	10.4 (10/96)	6 (6/100)	0.304
JO1 (%)	10.4 (10/96)	2 (2/100)	0.010
SSB (%)	26 (25/96)	15 (15/100)	0.076
RNP (%)	7.3 (7/96)	4 (4/100)	0.366
CENPB (%)	2.1 (2/96)	2 (2/100)	1.000
ANA (%)	19.8 (19/96)	14 (14/100)	0.341
ZP	11.5 (11/96)	8 (8/100)	0.474
ACA	1 (1/96)	1 (1/100)	1.000
MPO	2.1 (2/96)	1 (1/100)	0.615
PR3	14.6 (14/96)	2 (2/100)	0.001
TM	8.3 (8/96)	6 (6/100)	0.587
RIBP	10.4 (10/96)	8 (8/100)	0.626
RNPA	8.3 (8/96)	5 (5/100)	0.400
PCNA	3.1 (3/96)	3 (3/100)	1.000
dsDNA	2.1 (2/96)	0	0.239
HISTONES	1 (1/96)	0	0.490
GBM	4.2 (4/96)	3 (3/100)	0.717
RF	0	0	--

^#^The cutoff value was equivalent to the mean value for the serum assay controls (n = 100) plus 3SD (99% CI).

The level of positivity of anti-PR3 and anti-JO-1 observed in POI patients have been presented in figures (see the Additional files 2 and 3).

## Discussion

Variety of possible causes of POI reflects the heterogeneity of POI. None of the causes seems to predominate. However, their true incidence in the majority of the cases of POI remains unclear, so the scientists working in this area should be focus on the etiology of POI [9]. Autoimmunity appears to play a role in some cases of POI. The study shows that POI women group have significant higher levels of Jo-1 and PR3 antibodies (p = 0.010 and p = 0.001) whereas the circulation levels of ANAs, dsDNA, histone, RNP, Sm, Scl-70, CEN, ZP, AC, RF, GBM, PCNA, MPO and TM antibodies were similar between the two groups.

Autoimmune mechanisms are involved in the pathogenesis of up to 30% of cases in idiopathic POI [10]. It has been estimated that, in adult patients with POI, 18-30% also have an autoimmune disease and up to 50% have other evidence of autoimmunity [11]. The diagnosis of autoimmunity diseases relies on clinical, biological and histologic parameters. But in our study, all patients and healthy control women had no clinical symptom of autoimmunity disease. Detection of specific autoantibodies remains the most practical clinical research marker of any of autoimmune disease, so we selected a panel of well-established markers which are used in the diagnosis of autoimmunity diseases to investigate serologic autoimmunologic parameters in POI women. To our knowledge, no such data have been published in women with POI.

Human ovary is commonly the target of an autoimmune attack in cases of organ or non-organ specific autoimmune disorders leading to the ovarian dysfunction. Colafrancesco et al. described three clinical cases who developed premature ovarian failure following administration of the HPV vaccine [12]. Autoimmune etiology includes the presence of lymphocytic oophoritis, autoantibodies to ovarian antigens and associated autoimmune disorder. Anti-ovarian antibodies have been demonstrated in patients with SLE and primary Sogren’s syndrome and polyglandular syndrome [13]. Autoimmunity is an important mechanism for accelerated destruction of ovarian follicles.

Development of ovarian auto-antibodies is a causative factor in most POI cases. Although controversial, the presence of circulating antiovarian antibodies (AOA) may be considered a marker of autoimmune premature ovarian failure. Vallotton and Forbes were the first to describe the presence of antibodies from POI serum. Prevalence of antibodies in POI patients varied among investigators from 24%-73.3%. Different antibody test format, antigen preparation and criteria for different studies and several antigenic targets lead to different results [14-17]. Wheatcroft et al. reported an incidence ranging from 24-60%, depending on the source of ovarian antigen [2]. Besides, one of the major drawbacks in the detection of ovarian antibodies in serum is the higher rate of false positives. ZP is an extracellular matrix that surrounds the mammalian oocyte and plays an important role in normal folliculogenesis and fertilization, so ZP could be an important ovarian antigen [18,19]. Pires et al. indicated that several proteins from other cellular targets such as oocytes, corpus luteum, theca and granulose cells are also involved in ovarian autoimmunity [20]. In our study, with its strong immunogenicity and its possible relation with premature ovarian failure, we use the human ZP as antigen, but no difference was observed in the serum level of AOA between POI patients and control (11.5% & 8%).

Our data show that circulations level of PR3 and Jo-1 antibody are elevated in women with POI compared with healthy women. PR3 is the target of anti-neutrophil cytoplasm Abs (ANCA) in granulomatosis with polyangiitis, a form of systemic vasculitis [6,21]. In our study, among 96 POI patients, 14 patients have anti-PR3 higher levels, which 3 patients had histories of mumps, and the others have no any medical history. Anti-Jo-1 antibody, one of the aminoacyl tRNA synthetases antibodies, is associated with a more abrupt onset of fever, cracked hands, Raynaud’s phenomenon, interstitial lung disease, arthritis and PM/DM, which was reported in 20-30% of patients with PM/DM. Antibody to Jo-1 is a kind of anti-nuclear antibody. Anti-Jo-1 antibody levels correlated modestly with muscle and joint disease and more striking associations emerged in a smaller longitudinal subset of patients that link anti-jo-1 antibody levels to muscle, joint, lung, and global disease activity [22-24].there are 10 patients who had positive anti-Jo-1 antibody, 2 patients had histories of tuberculosis and 2 patients had histories of diabetes mellitus. The high level of PR3 and Jo-1 antibody suggest that a role for autoimmunologic process might be present in pathogenesis of POI. But the evaluation of the exact pathways of these antibodies remains to be determined.

Autoimmune diseases, such as autoimmune thyroiditis, diabetes mellitus type1, poparathyroidism, SLE, rheumatoid arthritis and Addison’s disease, frequently coexist with POI. 10-40% of women with POI have clinical autoimmune disease, the most common of these is hypothyroidism with an incidence of 27%, followed by diabetes mellitus (2.5%) and Addison’s disease (2.5%) [10,25]. In our study, 4 and 2 patients had histories of thyroid disorder and diabetes mellitus respectively whereas there was no thyroid disorder and diabetes mellitus in healthy-control women. Absence of clinical evidence of other endocrine autoimmune disease, POI patients often exhibit organ-specific auto-antibodies, but there were no significant in antibodies to dsDNA, histone, RNP, Sm, Scl-70, CEN, AC, GBM, PCNA, MPO, TM between the two groups (*P* > 0.05). Joe Feuerstein reported reversal of premature ovarian failure in a patient with Sjogren syndrome, laboratory tests showed a positive ANA, and a positive SS-A Sjogren antibody, positive anti-CCP and positive anti-Smith [26].

There are no convincing data at this time that patients with systemic autoimmune disorders such as SLE or rheumatoid arthritis (RA) have higher rates of POI than that of general population. But non-organ specific autoantibody has been reported in patients in POI, including antinuclear antibodies and rheumatoid factor [27]. ANA positivity of 42-77% has been reported among chromosomally normal women with POI, POI is not a well-described complication of systemic lupus erythematosis [4] and a positive rheumatoid factor. Interestingly, development of new onset SLE has been associated with slightly earlier age of natural menopause and early age at menopause may be a risk factor for development of rheumatoid arthritis. In our study, there is no significant in ANAs, RF between POI and healthy women groups.

Screening serologic tests, however, are not necessarily recommended because of high false-positive rates. Serologic tests are most useful in confirming or ruling out the diagnosis; for example, SLE is rarely present when ANAs are absent; the absence of RF will not rule out RA, but its presence confirms it. Polymyalgia rheumatica and RA, with an incidence of 1 in 2000 to 3000, are the most common autoimmune disorders. Other autoimmune diseases such as SLE, vasculitis, polymyositis, and dermatomyositis are seen infrequently in general practice. Menopause may affect disease prognosis and treatment.

Mumps oophoritis has been considered to be a cause of POF [28-30], in our study there are 9 patients who had mumps histories. 3 patients have positive PR3 and the others have no any positive antibody.

Our study shows that serum levels of Jo-1 and PR3 antibodies are elevated in women with POI compare with health controls. These results suggest that a role for autoimmunologic process might be present in pathogenesis of POI. Whether or not women with POI are at an increased risk for developing autoimmune diseases is unknown and should be investigated in future trials. The weaknesses of this study are the absence of a direct clinical implication and the necessity elucidating the possible pathways involved.

## Conclusions

This study shows that JO-1 and PR3 antibodies were significantly higher in POI women group and may be related with POI, which might help to determine the real prevalence of autoimmune POI. The interplay of autoimmunity with the reproductive endocrine system is complex and incompletely understood yet. We provide new proof on serologic autoimmunologic parameters in women with POI here. The evaluation of the exact pathways of these antibodies to POI remains to be determined.

## Abbreviations

POI: Primary ovarian insufficiency; POF: Premature ovarian failure; SLE: Systemic lupus erythematosus; TM: Thyroid microsomal; MPO: Myeloperoxidase; ACA: Adrenocortical antibodies; ANA: Antinuclear antibodies; RF: Rheumatoid factor; ZP: Zona pellucida; ds-DNA: Double-stranded DNA; ssDNA: Single-stranded DNA; RNP: Nuclear ribonucleoprotein; Sm: Smith antigen; SSA: Sjogren’s syndrome A; SSB/La: Sjogren’s syndrome B; PR3: Proteinase 3; ACNA: Anti-neutrophil cytoplasmic antibody; Scl-70: Scleroderma-associated antigen; PCNA: Proliferating cell nuclear antigen; GBM: Glomerular basement membrane; MCTD: Mixed connective tissue disease.

## Competing interests

The authors declare that they have no competing interests.

## Authors’ contributions

All authors declare to have participated in the study as mentioned and approved the final version. XZ, JQ were involved in study design, XZ, RL, LW, and PL were involved in acquisition of data and contributed to analysis and interpretation of data. XZ contributed to drafting the manuscript. XZ, JQ, RL, LW, and PL contributed to critical revision of manuscript for important intellectual content. JQ had full access to all the data in the study and had final responsibility for the decision to submit for publication.

## Supplementary Material

Additional file 1 Ethics statement approved by the Peking University Third Hospital ethical committee. Click here for file

Additional file 2 The levels of anti-PR3 antibody observed in POI patients and compared healthy women. Click here for file

Additional file 3 The levels of anti-Jo1 antibody observed in POI patients and compared healthy women. Click here for file
