# Supplementary figures and images for: Serologic autoimmunologic parameters in women with primary ovarian insufficiency
Source: BMC Immunol. 2014 Mar 10;15:11. doi: 10.1186/1471-2172-15-11 (PMC4016123; doi:10.1186/1471-2172-15-11)

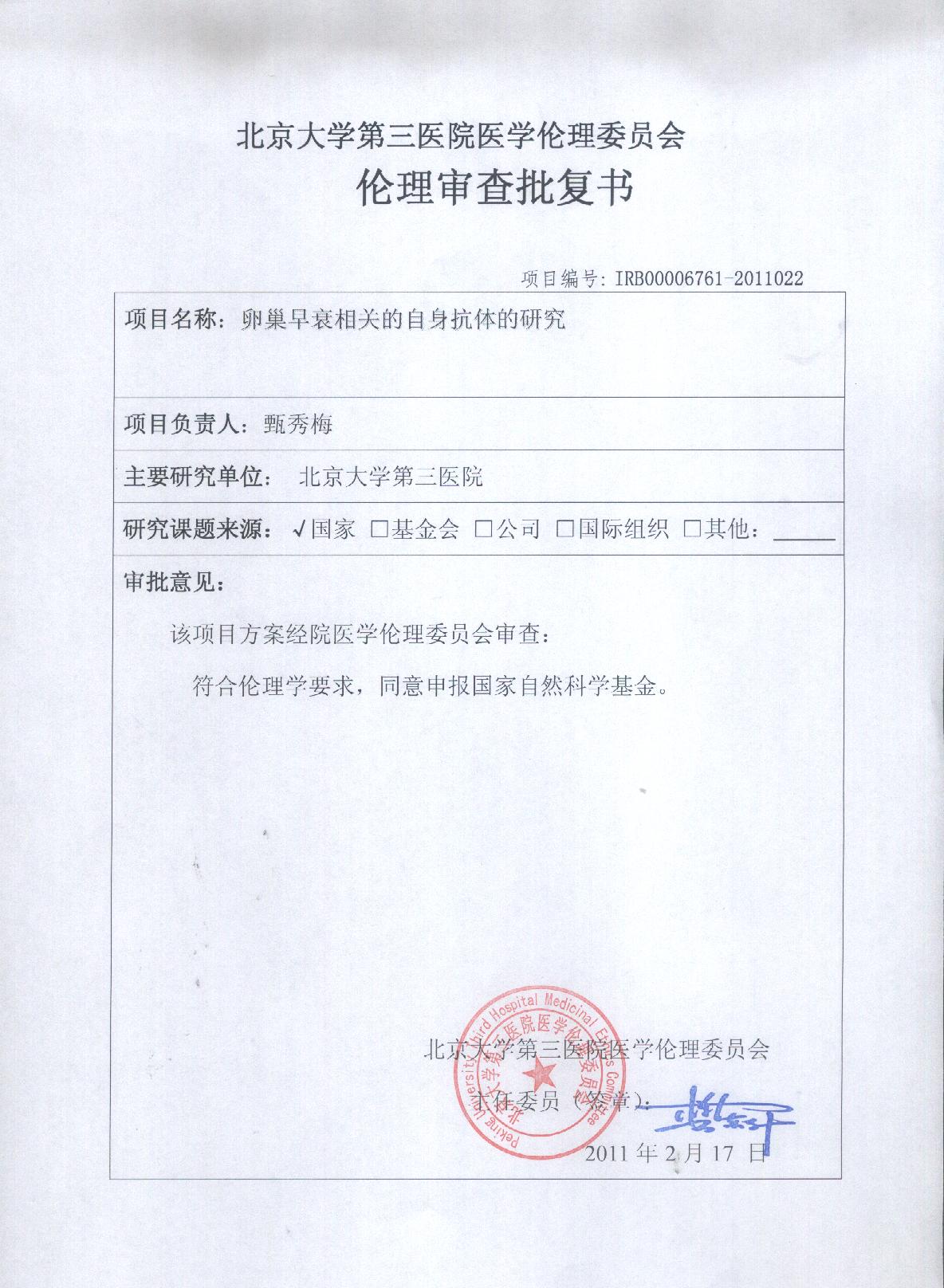

Supplement: Additional file 1 — Ethics statement approved by the Peking University Third Hospital ethical committee. [file 1471-2172-15-11-S1.jpeg]

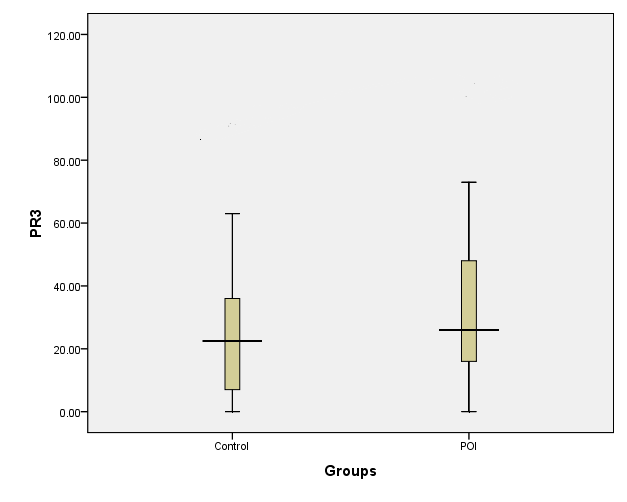

Supplement: Additional file 2 — The levels of anti-PR3 antibody observed in POI patients and compared healthy women. [file 1471-2172-15-11-S2.bmp]

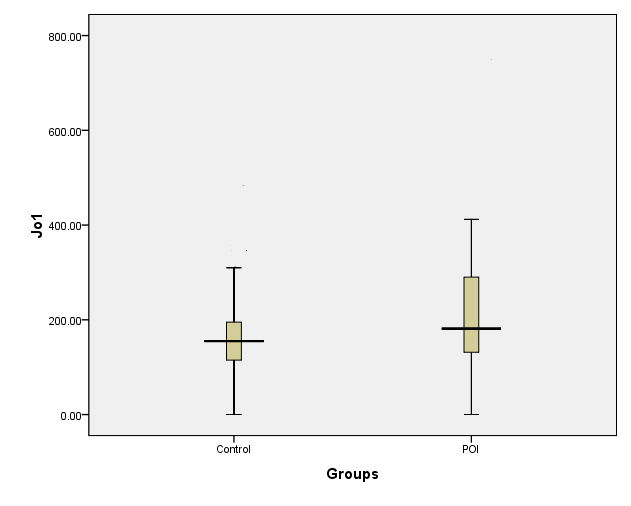

Supplement: Additional file 3 — The levels of anti-Jo1 antibody observed in POI patients and compared healthy women. [file 1471-2172-15-11-S3.bmp]
